# Evaluating an online self-compassion-based mindfulness course for mothers of autistic children

**DOI:** 10.3389/fpsyg.2026.1761742

**Published:** 2026-04-17

**Authors:** Jacob Barnum, Maddie Blackham, Maya Hollingshead, Matthew Hatch, Sarah Wilkinson, Madeline Gillies, Rebecca A. Lundwall

**Affiliations:** Psychology Department, Brigham Young University, Provo, UT, United States

**Keywords:** autism, mindfulness, parenting daily hassles, parenting stress, self-compassion

## Abstract

Self-compassion-based mindfulness training has been associated with improved psychological well-being. This study examined whether an established online self-compassion course was associated with changes in self-compassion, flourishing, parenting stress, parenting daily hassles, and family quality of life among mothers of infants, including mothers of autistic children. Mothers were grouped based on whether they had an autistic child or not. We assessed baseline differences prior to course enrollment, pre–post changes among participants who completed the course, and whether changes differed by group. At baseline, 67 mothers completed at least three questionnaires. Between 27 and 44 mothers (depending on the measure) completed both pre- and post-course assessments. At baseline, mothers of autistic children reported lower family quality of life and higher parenting stress and daily hassles than comparison mothers. Among course completers, self-compassion and flourishing increased from pre- to post-course. No significant group × time interactions were observed. Parenting stress, daily hassles, and family quality of life did not show detectable changes over time. Findings are preliminary and non-causal due to the absence of a control group. Results are consistent with prior research indicating lower baseline well-being among mothers of autistic children and suggest that online self-compassion training may support internal coping processes regardless of autism status. However, some caregiving demands appear stable despite improvements in self-compassion.

## Introduction

Parenting takes place within service and support systems that vary significantly in accessibility, coordination demands, and resource availability. For some families—particularly those raising autistic children—logistical and structural demands such as multiple appointments, limited respite, and long waitlists can elevate daily caregiving load and limit opportunities for parental well-being.

Research consistently shows that parents of autistic children report higher stress and more frequent daily hassles than parents of non-autistic children (Hastings et al., 2005; Craig et al., 2016; Tehee et al., 2009). This is not necessarily because autistic children themselves are a burden, but likely because there are more societal demands on parents when they have autistic children (Gülbetekin et al., 2024; Rusu et al., 2024).

Nevertheless, stress-management techniques have been linked to parent well-being (Lindo et al., 2016). Mindfulness refers to present-moment, nonjudgmental awareness of internal and external experiences (Bishop et al., 2004). Although mindfulness is often taught alongside meditation practices, mindfulness itself refers to a state of awareness rather than a specific technique (Creswell, 2017). The type of mindfulness we chose to use includes self-compassion.

Neff (2003, 2023) describes self-compassion as including three components—self-kindness, common humanity, and mindful awareness—and as being associated with reduced stress and improved coping in caregiving contexts. For parents navigating systemic pressures, self-compassion may buffer against internalized stigma, self-blame, and chronic stress (Gülbetekin et al., 2024; Jefferson et al., 2020; Rusu et al., 2024; Ozturk and Güzel, 2025). Overall, self-compassion training has a strong evidence base for improving psychological well-being, and online formats reduce barriers to participation for parents managing intensive caregiving schedules.

The present study examined whether an established online self-compassion-based mindfulness course would replicate known emotional benefits among parents of infants, including those raising autistic children. We expected baseline group differences in system-related stress, overall gains in self-compassion and flourishing for both groups, and potentially larger gains among parents of autistic children.

## Methods

### Participants

We used a longitudinal pre-post design to examine changes associated with participation in the self-compassion-based mindfulness course. We recruited participants through autism support networks in the Intermountain West, including community organizations, social media groups, and school-based channels. Recruitment materials invited parents of infants aged 8 to 23 months to participate in a study evaluating an online self-compassion program. We focused on this age range because some parents report more difficulty parenting as their children become more active (Crnic and Booth, 1991) and because it is in line with other research we have conducted (Lundwall, 2019; ManyBabies Consortium, 2020; Zettersten et al., 2024).

Eligibility criteria required participants to (a) be the parent of at least one infant between 8 and 23 months old and (b) reside in the Intermountain West. We categorized participants into two groups: (1) parents of autistic children, defined as having at least one formally diagnosed or provider-suspected autistic child in addition to the target infant; and (2) a comparison group, defined as reporting no known autistic relatives within three degrees. Due to reduced interest, we did not enroll a control group that did not complete mindfulness. A total of 71 parents expressed interest in the study; 67 completed the questionnaires for the baseline comparison (Table 1). All of these 67 parents identified as mothers.

**Table 1:** Demographics at baseline (*N* = 67).

Variable	Parents with a child with ASD	Parents without a child with ASD
Average baseline questionnaires	4.28 (0.59)	4.61 (0.50)
Percent female parent	100%	100%
Mean parent age in years (*SD*)	33.59 (5.69)	29.66 (7.02)
Percent Caucasian	93%	97%
Average children 0–12 years in home	3.15 (1.13)	2.11 (1.10)
Average children 13–18 years in home	0.25 (0.61)	0.16 (0.50)
Infant % female	43%	48%
Infant age (months)	12.22 (2.13)	13.62 (4.96)

Participants were required to have a child within the 8–23-month range (this would be the younger sibling of an autistic child for the autistic group, but for the non-autistic group, the child did not necessarily need to have an older sibling). Mothers of autistic children had an average of 3.53 children (*SD* = 1.55, range = 2 to 7 children) between 0 and 18 years old. Mothers without autistic children had an average of 2.31 children (*SD* = 1.39, range = 1 to 7 children) between 0 and 18 years old.

### Materials

#### Self-compassion-based mindfulness course

The Gift of Self-Compassion (Warren, 2020; Linford and Warren, 2024) is an asynchronous, web-based self-compassion-based mindfulness course designed to cultivate self-kindness, common humanity, and mindful awareness. The course includes instructional videos, guided mindfulness and compassion exercises, reflective writing prompts, and brief psychoeducational readings. Mothers accessed materials through a secure website and used a companion workbook containing written exercises and prompts.

The course structure emphasizes applying self-compassion in everyday contexts, including navigating stress, responding to difficult internal experiences, and reframing self-critical thoughts. Modules include: (1) course introduction, (2) fundamentals of self-compassion, and (3) the practice of self-compassion. Mothers progressed at their own pace, and we encouraged them to engage with the materials for brief intervals each day.

### Measures

#### Family quality of life

We used the Beach Family Quality of Life Scale (Hoffman et al., 2006) to assess satisfaction across family interaction, parenting, psychological well-being, physical/material well-being, and disability-related support. Items are rated on a five-point Likert scale. Internal consistency estimates are high (*α* = 0.88–0.94; Hoffman et al., 2009).

#### Flourishing

The Survey on Flourishing (SURF) is a 19-item self-report scale measuring multiple aspects of well-being, including satisfaction, affect, and meaning (Linford and Warren, 2022). Items are rated on a five-point scale. Internal consistency is high (*α* = 0.95). SURF scores correlate positively with the PERMA-Profiler and Satisfaction with Life Scale.

#### Parenting daily hassles

Parenting daily experiences were assessed with the Parenting Daily Hassles Index (PDH; Crnic and Greenberg, 1990). Mothers rated the frequency and perceived intensity of 20 common caregiving hassles. Frequency ratings use a four-point scale; intensity ratings use a five-point scale. Higher scores reflect more frequent or more intense hassles. Internal reliability is strong (*α* = 0.87; Crnic and Booth, 1991).

#### Parenting stress

Parenting stress was measured using the Parenting Stress Index-Short Form (PSI-4-SF; Abidin, 2012). This 36-item measure includes three domains: parental distress, parent-child dysfunctional interaction, and difficult child. Normative scoring indicates typical stress between the 15th and 80th percentiles. Internal reliability for the total score is high (*α* = 0.91; Abidin, 2012).

#### Self-compassion

Self-compassion was measured with the MBS101-Self-Compassion scale (Linford and Warren, 2022), a 12-item self-report instrument assessing self-kindness, common humanity, and mindful awareness of emotional experiences. Items are rated on a five-point Likert scale. Internal consistency is high (*α* = 0.91), with strong convergent validity with the Neff Self-Compassion Scale (*r* = 0.85).

### Procedure

After baseline measurement (prior to the option to enroll in mindfulness), the study consisted of two timepoints: a pre-course assessment timepoint and a post-course assessment timepoint. Mothers provided informed consent according to IRB #X2019-427-BYU and the Declaration of Helsinki. Mothers then completed online questionnaires assessing family quality of life, flourishing, parenting daily hassles, parenting stress, and self-compassion. Mothers received access to the self-compassion-based mindfulness course and the accompanying workbook after completing the pre-course questionnaires. They completed the course in an average of 39.42 weeks (*SD* = 12.66). This gap was longer than intended and introduces the possibility of maturation effects which we cannot address without a control group.

The self-compassion course comprises three modules (introduction, fundamentals of self-compassion, and practice of self-compassion), delivered through brief videos, guided exercises, and reflective prompts. We encouraged mothers to engage with course materials for brief daily intervals (10–15 min) over at least 8 weeks. Research assistants contacted mothers every 7–10 days to provide procedural support, clarify technical questions, and encourage continued engagement.

At the conclusion of the course window, mothers completed the same set of questionnaires. Mothers also completed a brief feedback survey regarding their experience with the course. All mothers received compensation upon completing the post-course measures.

For analytic clarity, “baseline” refers to the first assessment completed by all participants, regardless of whether they ultimately enrolled in the mindfulness course. “Pre-course” refers specifically to the assessment immediately preceding course access among those who completed the intervention.

### Statistical analyses

Analyses were conducted in *IBM SPSS Statistics* (Version 28.0). To assess pre- and post-mindfulness differences between mothers of autistic children and the comparison group, we used independent *t-*tests to compare pre-course scores on all measures. Group status served as the independent variable. Due to the exploratory nature of this study, we did not correct for multiple comparisons.

To evaluate changes from pre-course to post-course for all mothers who completed both assessments, we conducted paired *t-*tests. These analyses tested whether participation in the self-compassion course was associated with changes in self-compassion, flourishing, parenting daily hassles, parenting stress, and family quality of life.

Finally, we used repeated-measures ANOVAs to examine group × timepoint interactions, testing whether changes over time differed between mothers of autistic children and the comparison group.

## Results

### Baseline differences

A total of 67 mothers contributed data to the study at baseline: 29 mothers of autistic children (mother’s age *M* = 33.59 years, *SD* = 5.69 years; 100% female) and 38 parents in the comparison group (mother age *M* = 29.66 years, *SD* = 7.02 years; 100% female).

Independent *t-*tests compared pre-course scores between groups. We expected differences related to system-level demands experienced by mothers of autistic children.

Consistent with expectations, the group for mothers with autistic children reported significantly higher parenting daily hassles than the comparison group (*p* < 0.001, Cohen’s *d* = −0.93) and higher parenting stress (*p* = 0.002, Cohen’s *d* = −1.26). They also reported significantly lower family quality of life (*p* = 0.04, Cohen’s *d* = 0.51). No significant group differences emerged for flourishing or self-compassion. See Table 2 for comparisons prior to enrollment in mindfulness.

**Table 2:** Baseline questionnaire scores.

Measure	Autism group *M* (*SD*)	*n*	Comparison *M* (*SD*)	*n*	*t*-statistic	df	*p*-value	Cohen’s *d*
Family quality of life	96.72 (14.24)	29	103.00 (10.58)	38	2.07	65	0.04^*^	0.51
Flourishing	96.00 (10.50)	28	95.03 (10.52)	38	−0.37	64	0.71	−0.09
Parenting daily hassles	53.46 (13.01)	28	41.42 (9.81)	38	−4.29	64	<0.001^*^	−0.93
Parenting stress^a^	100.40 (30.23)	10	70.83 (17.47)	23	−3.32	31	0.002^*^	−1.26
Self-compassion	51.41 (9.39)	29	51.74 (11.27)	38	0.12	65	0.90	0.03

We assumed Cohen’s benchmarks for effect size as *d* = 0.20 = small, *d* = 0.50 = medium, and *d* = 0.80 to be large (Cohen, 1988). Negative Cohen’s *d* values for those measures with *p* < 0.05 indicate higher scores for those parents with an autistic child.

We also compared those who completed the mindfulness course to those who did not on demographic variables (those in Table 1). Those who completed the intervention completed more baseline questionnaires than those who did not complete the intervention [*t*(65) = −2.92, *p* = 0.005]. No other demographic variable differences were significant.

### Changes from pre-course to post-course

A total of 51 mothers completed the self-compassion course and completed at least three questionnaires. This comprised 20 mothers of autistic children (mother age *M* = 34.58 years, *SD* = 5.77 years) and 31 mothers in the comparison group (mother age *M* = 30.10 years, *SD* = 7.27 years). However, mothers did not always complete all paired (pre-course and post-course) questionnaires. Overall, there were between 27 and 44 completions, depending on the questionnaire (see Table 3).

**Table 3:** Combined groups changes pre-course and post-course.

Measure	Mean	*SD*	*n*	*t*-statistic	df	*p*-value	Cohen’s *d*
Family quality of life_1_	100.85	10.72					
Family quality of life_2_	101.26	10.15					
Diff.	0.41		39	−0.29	38	0.77	−0.05
Flourishing_1_	93.43	11.63					
Flourishing_2_	95.91	8.56					
Diff.	2.48		44	−2.13	43	0.04^*^	−0.32
Parenting daily hassles_1_	46.26	11.66					
Parenting daily hassles_2_	43.32	11.28					
Diff.	−2.95		38	2.00	37	0.05	0.32
Parenting stress_1_	83.63	25.24					
Parenting stress_2_	79.33	11.63					
Diff.	−4.30		27	1.69	26	0.10	0.32
Self-compassion_1_	51.23	10.22					
Self-compassion_2_	56.23	11.61					
Diff.	5.00		44	−3.60	43	<0.001^*^	−0.54

Negative Cohen’s *d* values indicate the comparison group scored higher than the group for mothers with autistic children. We assumed Cohen’s benchmarks for effect size as *d* = 0.20 = small, *d* = 0.50 = medium, and *d* = 0.80 to be large (Cohen, 1988).

Across both groups, participation in the self-compassion course was associated with significant increases in self-compassion (*p* < 0.001, Cohen’s *d* = 0.54) and flourishing (*p* = 0.04, Cohen’s *d* = −0.32). Scores for family quality of life (*p* = 0.77, Cohen’s *d* = −0.05) and parenting stress (*p* = 0.10, Cohen’s *d* = 0.32) did not show statistically significant changes. Parenting daily hassles showed a small decrease (Cohen’s *d* = 0.32); the associated *p*-value (0.05) suggests this effect should be interpreted cautiously.

These findings indicate that the self-compassion-based mindfulness course was associated with improvements in psychological well-being (self-compassion, flourishing), but effects on system-related stressors and family-level outcomes were not statistically detectable within the current sample (see Table 3).

### Group × timepoint interactions

**Figure 1:**
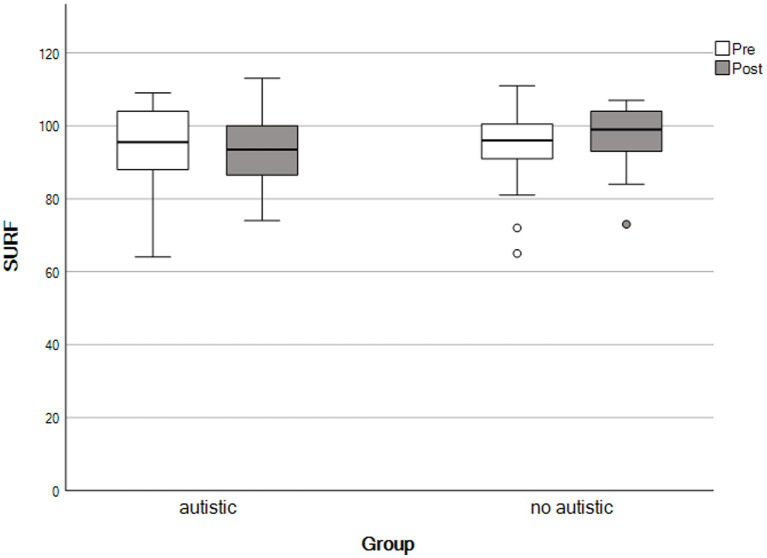
Family quality of life scores by time and group. The X-axis represents parents with an autistic child and parents whose child had no autistic relatives.

**Figure 2:**
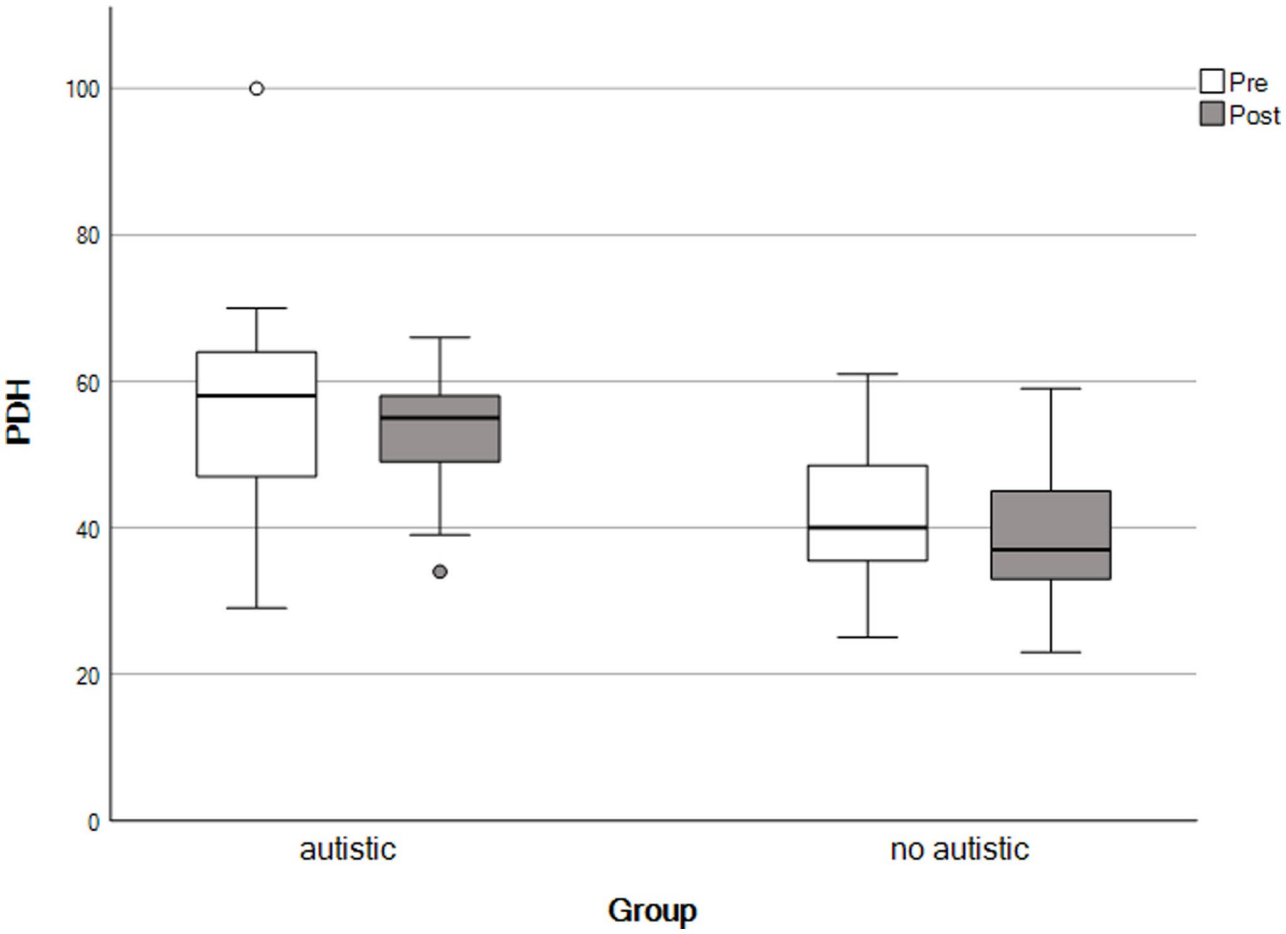
Self-compassion scores by time and group. The X-axis represents parents with an autistic child and parents whose child had no autistic relatives.

**Figure 3:**
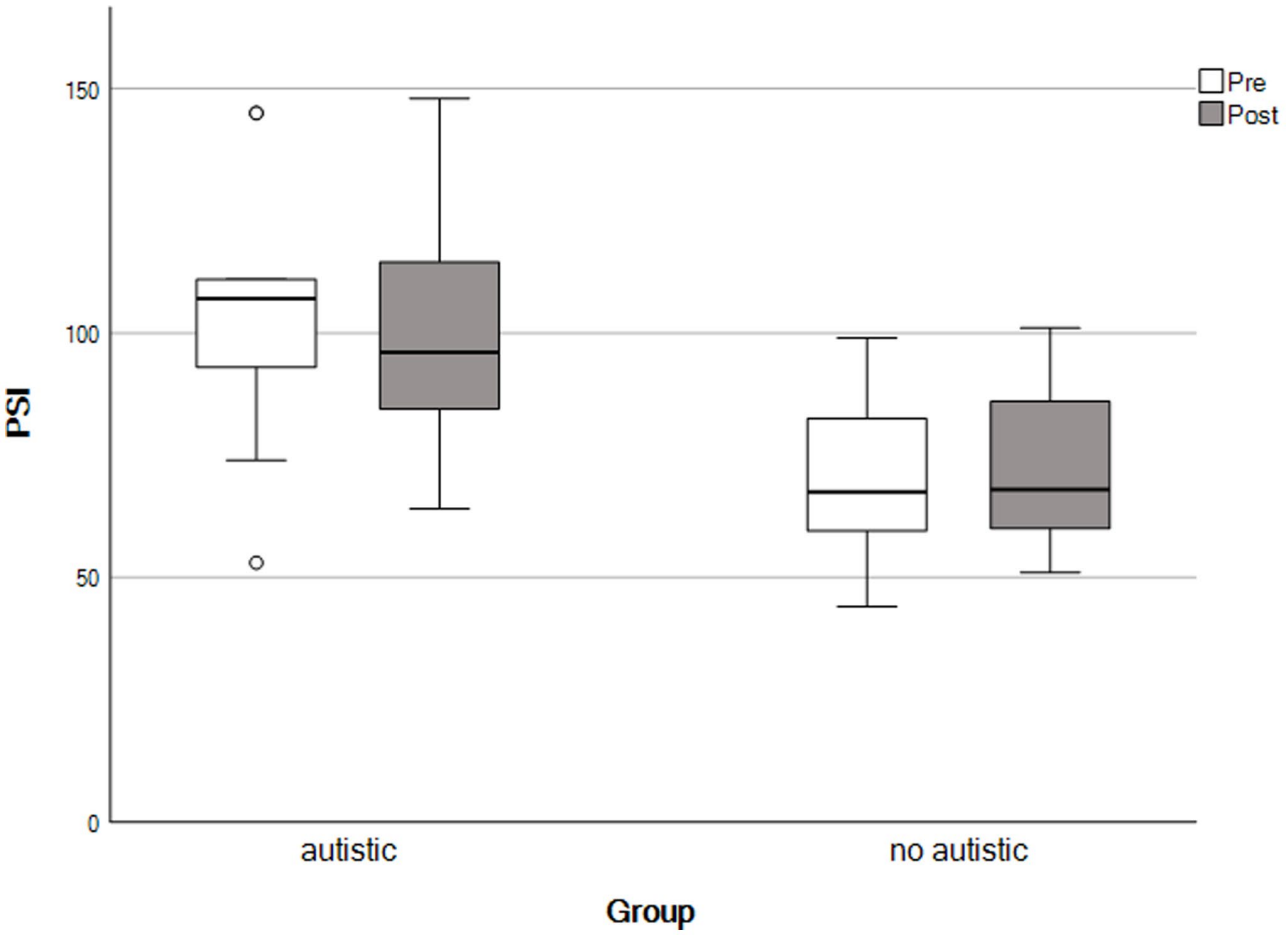
Parenting daily hassles scores by time and group. The X-axis represents parents with an autistic child and parents whose child had no autistic relatives.

**Figure 4:**
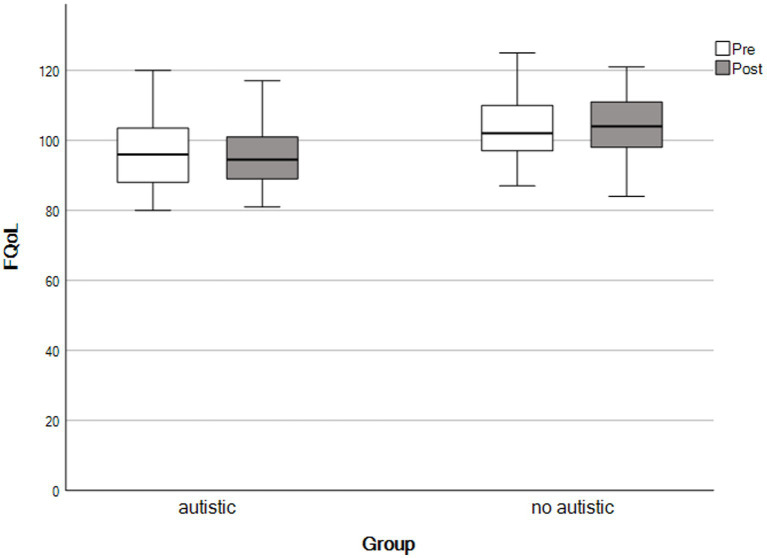
Parenting stress scores by time and group. The X-axis represents parents with an autistic child and parents whose child had no autistic relatives.

**Figure 5:**
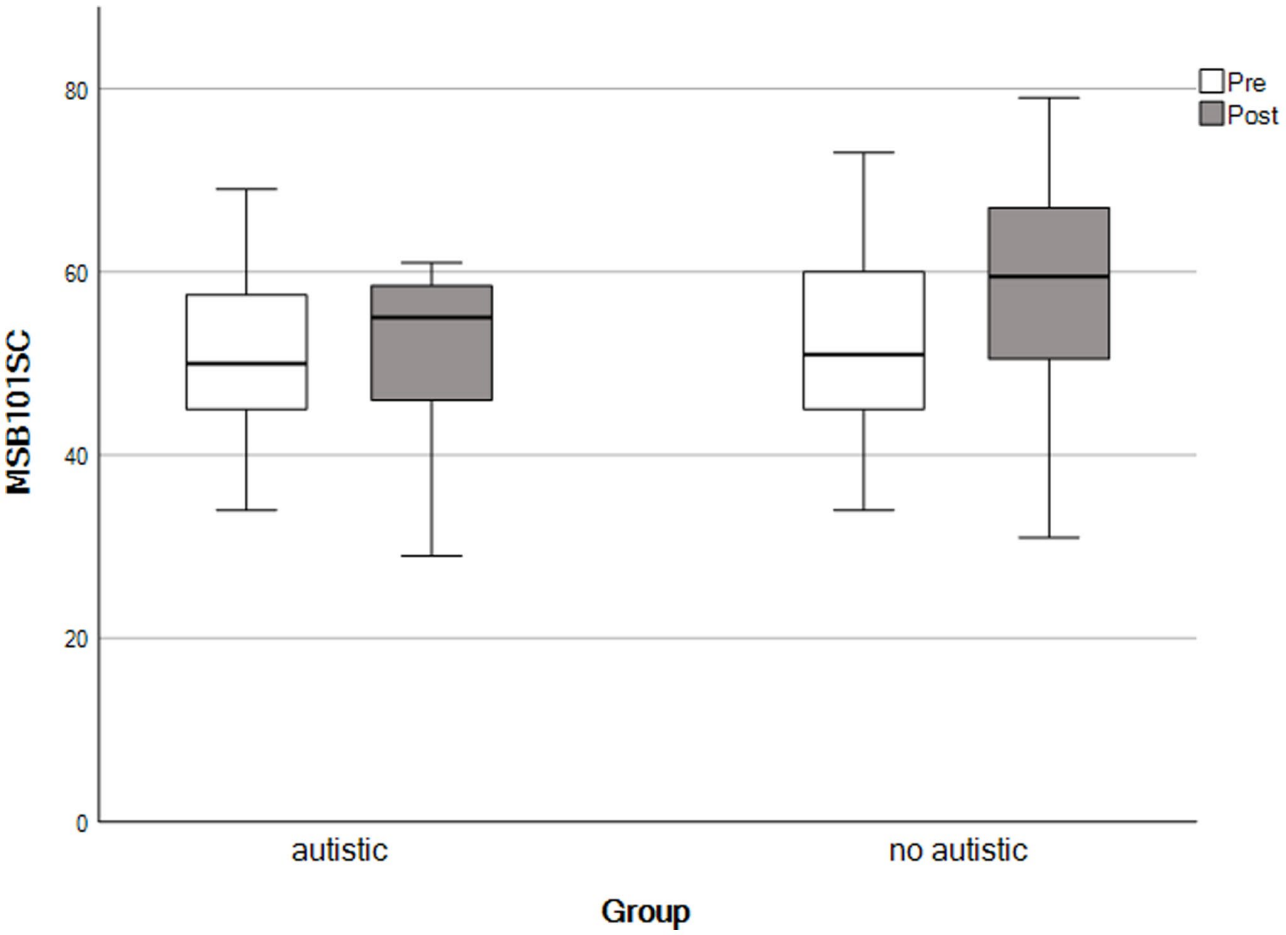
Flourishing scores by time and group. The X-axis represents parents with an autistic child and parents whose child had no autistic relatives.

For the final analysis, the same mothers contributed data as for the paired samples *t*-tests, above. We used repeated-measures ANOVAs to detect group × time interactions. We observed no significant interaction effects across any outcomes, consistent with both groups showing similar patterns of change following the self-compassion course (see Table 4 and Figures 1–5).

**Table 4:** Interactions between groups (have autistic children vs. comparison group) and timepoint (pre-course to post-course).

Effect	df_1_	df_2_	*F*	*p*-value	*η* ^2^_p_
Family quality of life					
Group	1	37	5.87	0.02	0.14
Timepoint	1	37	0.005	0.95	<0.001
Group × timepoint	1	37	0.56	0.46	0.02
Flourishing					
Group	1	42	0.94	0.34	0.02
Timepoint	1	42	3.34	0.08	0.07
Group × timepoint	1	42	0.63	0.43	0.02
Parenting daily hassles					
Group	1	36	16.17	<0.001	0.31
Timepoint	1	36	2.87	0.10	0.07
Group × timepoint	1	36	0.32	0.57	0.01
Parenting stress					
Group	1	25	23.43	<0.001	0.48
Timepoint	1	25	4.63	0.04	0.16
Group × timepoint	1	25	2.02	0.17	0.08
Self-compassion					
Group	1	42	2.72	0.11	0.06
Timepoint	1	42	9.51	0.004	0.18
Group × timepoint	1	42	2.65	0.11	0.06

We assumed Cohen’s benchmarks for effect size as *d* = 0.20 = small, *d* = 0.50 = medium, and *d* = 0.80 to be large (Cohen, 1988).

### Course engagement and participant feedback

Forty-six mothers completed the feedback survey and reported engaging in course practices on an average of 18 days (range = 4–35), with a mean of 10 min per day (range = 3–30). Completion patterns varied (20 of the 29 mothers with an autistic child who began the study before enrolling in mindfulness and 31 of the 38 of the comparison group completed mindfulness). We used a chi-square test of independence to determine if the proportion of those who completed mindfulness was higher in one group versus the other. We found no statistically significant difference [*χ*^2^ (1, *N* = 67) = 1.44, *p* = 0.23, *φ* = 0.15]. The effect size here represents a small, imprecisely estimated difference unable to confirm any differences in completion rates per group.

Among those who completed the survey, 38 people provided positive feedback. The most frequent comments (47%) relate to guided practice time. Another 29% emphasized the knowledge they had gained. Thirty-one people also provided negative feedback. The most common complaint was that the course was time-consuming (53%). The next most common complaint was that some of the activities felt uncomfortable (21%). Twenty-four percent said they would not have changed the course.

## Discussion

### Main findings

Consistent with prior research, participation in the self-compassion course was associated with increased self-compassion and flourishing. These findings replicate known benefits of self-compassion training (Cengiz and Kiliҫ, 2025; Fenning et al., 2024; Jefferson et al., 2020; Neff and Faso, 2015; Rayan and Ahmad, 2017) and suggest that even modest engagement may support well-being. Importantly, we observed improvements even though many system-level stressors remained unchanged. It cannot be concluded, however, that the course caused the increase in self-compassion and flourishing due to the lack of a control group that did not complete mindfulness.

Externally shaped outcomes such as daily hassles, and family quality of life did change significantly, highlighting the potential for self-compassion to support internal coping without modifying structural caregiving demands, which we did not measure directly. However, self-compassion may help mothers relate to their experiences with greater warmth and balance.

### Between-group differences prior to the course

As expected, mothers of autistic children reported higher parenting daily hassles, parenting stress, and lower family quality of life at baseline. These differences may reflect broader caregiving demands frequently reported in the literature among parents of autistic children (Carbone et al., 2010; Hyman and Johnson, 2012; MacKenzie et al., 2023).

Notably, baseline self-compassion and flourishing levels did not differ between groups, suggesting that well-being may be less dependent on contextual care demands than daily logistical stressors (Crnic and Greenberg, 1990).

### Absence of interaction effects

The lack of interaction effects indicates that changes over time were similar for mothers in both groups, suggesting that the course may provide support that is generalizable across caregiving contexts. This suggests that the course may support general resilience (Cengiz and Kiliҫ, 2025; Jefferson et al., 2020) across diverse caregiving contexts. This interpretation aligns with self-compassion theory: the core skills—self-kindness, common humanity, and mindful awareness—are broadly applicable and not diagnosis-specific (Neff, 2003, 2023). However, it is also possible that earlier pre-post mindfulness differences represent maturational changes rather than the effectiveness of the course.

### Implications

The findings highlight that:

1. Structural and systemic demands may contribute to the higher parenting daily hassles reported by mothers of autistic children.
2. Online, flexible delivery formats may help mothers engage in emotional skill building despite time limitations.
3. The course was associated with enhanced well-being even when external circumstances remain unchanged.
4. Increased flourishing and self-compassion may reflect improved coping with stigma, uncertainty, or internalized expectations.

Because many external demands remain substantial, improvements in emotional well-being may not immediately translate into reductions in parenting stress or family quality of life. Future interventions may benefit from integrating self-compassion practices with practical supports to address both internal coping and external caregiving demands.

### Limitations and future directions

#### Study methodology

The study was designed to analyze changes in mental health variables through the use of a self-compassion intervention. However, as both of our groups completed the intervention, there was not a control group who did not participate in any intervention. Thus, any observed changes cannot be definitively attributed to the effect of the intervention (e.g., they could signal maturation effects). Other variables, including the natural passage of time, may have contributed to the results, preventing researchers from concluding there was a causal relationship between the intervention and the observed effects.

#### Course completion and engagement

A substantial proportion of mothers did not complete the full self-compassion-based mindfulness course, with nearly 25% discontinuing before finishing all modules. While this pattern is common in online interventions (Eysenbach, 2005; Linardon et al., 2025), it may have limited the magnitude of observed effects. In line with this idea, some reported that course requirements were difficult to fit into their schedules. This variation in exposure introduced heterogeneity in intervention dosage.

Given the extensive time demands on mothers of autistic children, it is not surprising that consistent engagement with a structured program presented challenges. These contextual realities highlight why flexible, modular online programs remain important, even when full completion is difficult for many families. Nevertheless, our observed improvements in self-compassion and flourishing suggest that even partial engagement may offer meaningful benefit.

#### Family structure and demographic factors

Family composition and size varied across mothers. Although previous research shows that family size, socioeconomic context, and family structure can influence parental stress and well-being (Hong and Liu, 2021; McAuliffe et al., 2017), the current study prioritized inclusive recruitment to ensure adequate sample size. As a result, we were not willing to exclude families based on family size. Future work could use more targeted recruitment or statistical modeling to examine how demographic factors shape both system-level burdens and responses to self-compassion practices. We should also note that, since no fathers completed the mindfulness course, our study cannot generalize to fathers.

#### Power considerations

The study’s sample size and incomplete questionnaire data limited statistical power, particularly for detecting small effects and group × timepoint interactions. Effect sizes for between-group differences varied from |0.09 to 1.23| (Table 2), although the parenting stress index included relatively small cell sizes in the autism group, which may reduce the stability of that estimate.

In the pre-post analyses, several outcomes were associated with small-to-moderate effect sizes, regardless of statistical significance. This pattern suggests that some non-significant findings may reflect limited power rather than the absence of meaningful change. Replication with a larger sample and more complete data would help clarify the magnitude and reliability of these effects.

No group × timepoint interaction terms reached statistical significance (Table 4). Given the modest sample size, the study may have been underpowered to detect small interaction effects; therefore, the absence of significant interactions should be interpreted cautiously.

#### Geographical context

The sample was drawn exclusively from mothers residing in the Intermountain West, a region with distinct geographic, demographic, and service-delivery characteristics. Although there is no direct evidence suggesting that parenting processes operate differently in this region, prior research indicates that parenting practices and parent–child associations can vary across U.S. regions (McKinney and Brown, 2017; McKinney et al., 2018). In addition, families in the Intermountain West may encounter unique contextual factors, including greater travel distances for specialty services and variability in service access between rural and urban areas. These structural conditions could influence intervention engagement or perceived outcomes and may differ from those in more densely resourced regions. Future research using multisite or nationally distributed samples would help clarify the extent to which service-system characteristics, cultural context, and socioeconomic variability shape mothers’ experiences and intervention engagement.

#### Qualitative insight

Mothers completed a brief post-course feedback survey, but we conducted no qualitative interviews. Interviews could have provided deeper insight into how mothers applied the self-compassion course in their daily routines, how they perceived systemic stressors, and which elements of the program felt most meaningful. Future research could integrate qualitative methods to capture a richer understanding of how self-compassion skills intersect with the structural challenges faced by families of autistic children.

#### Intervention scope and practical supports

This study focused on a self-compassion-based approach. Although participation in the course was associated with improvements in some well-being outcomes, practical supports are likely necessary to reduce externally imposed stressors. Future interventions might combine self-compassion practices with community-based resources to address both emotional and contextual demands.

## Conclusion

This study replicates established associations between online self-compassion training and improved internal coping processes in mothers of infants. Improvements were comparable for mothers of autistic and non-autistic children, suggesting the course may offer broadly applicable support rather than autism-specific effects. Because all participants received the intervention, findings should be interpreted as preliminary and non-causal.

The present findings suggest that self-compassion skills may help mothers relate to these demands with greater emotional balance and resilience. Because structural caregiving demands are unlikely to change through mindfulness practices alone, integrating psychological and contextual supports may be beneficial.

Future research should examine how self-compassion interventions can be integrated with community-based supports to address both internal coping and external caregiving demands. Strengthening emotional resilience may represent one component of multifaceted support for mothers navigating diverse caregiving contexts.

## Data Availability

The raw data supporting the conclusions of this article will be made available by the authors, without undue reservation.
